# INTERRUPTED TIME SERIES ANALYSIS OF THE 2015 DUTCH LONG-TERM CARE: ACUTE HOSPITAL UTILIZATION OF OLDER ADULTS

**DOI:** 10.1093/geroni/igad104.2250

**Published:** 2023-12-21

**Authors:** Joost Wammes, Pieter Bakx, Bram Wouterse, Bianca Buurman, Terrence Murphy, Janet MacNeil Vroomen

**Affiliations:** Amsterdam Medical Centers, Amsterdam, Noord-Holland, Netherlands; Erasmus University Rotterdam, Rotterdam, Noord-Holland, Netherlands; Erasmus University Rotterdam, Rotterdam, Zuid-Holland, Netherlands; Amsterdam Medical Centers, Amsterdam, Noord-Holland, Netherlands; Penn State University, State College, Pennsylvania, United States; Amsterdam Medical Centers, Amsterdam, Noord-Holland, Netherlands

## Abstract

In 2015, the Dutch government implemented a long-term care (LTC) reform primarily designed to promote older adults (≥65 years old) to age-in-place. Increased proportions of older adults living in the community may have resulted in more and longer acute hospitalizations. We evaluated the reform’s association with the monthly rate of acute clinical hospitalization and monthly average hospital length of stay (LOS), using an interrupted time series design of national hospital data (2009 to 2018) that controlled for population growth and seasonality, and calculated adjusted incident rate ratios (IRR). The pre-reform trend of hospitalization rate was increasing (IRR 1.002, 95% CI 1.001-1.002). A positive average reform effect was observed (IRR 1.116, 95% CI 1.070-1.165), accompanied by a negative change in trend (IRR 0.996, 95% CI 0.995-0.998). This resulted in a decreasing trend over the post-reform period (IRR 0.997, 95% CI 0.997-0.999). The pre-reform trend of LOS was decreasing (IRR 0.998, 95% CI 0.997-0.998), and the 2015 reform exhibited a positive change in trend (IRR 1.002, 95% CI 1.002-1.003). This resulted in a stabilization of LOS in the post-reform period (IRR 0.999, 95% CI 0.999-1.00). Our findings suggest that the increase in the rate of acute hospitalization after the reform implementation was temporary, whereas the LOS appeared to be longer than expected.

